# Ataxic Symptoms in Huntington’s Disease Transgenic Mouse Model Are Alleviated by Chlorzoxazone

**DOI:** 10.3389/fnins.2020.00279

**Published:** 2020-04-03

**Authors:** Polina A. Egorova, Aleksandra V. Gavrilova, Ilya B. Bezprozvanny

**Affiliations:** ^1^Laboratory of Molecular Neurodegeneration, Peter the Great St. Petersburg Polytechnic University, St. Petersburg, Russia; ^2^Department of Physiology, University of Texas Southwestern Medical Center, Dallas, TX, United States

**Keywords:** ataxia, cerebellum, Huntington’s disease, transgenic mice, chlorzoxazone

## Abstract

Huntington’s disease (HD) is a hereditary neurodegenerative disease caused by a polyglutamine expansion in the huntingtin protein, Striatum atrophy in HD leads to a progressive disturbance of psychiatric, motor, and cognitive function. Recent studies of HD patients revealed that the degeneration of cerebellum is also observed independently from the striatal atrophy during early HD stage and may contribute to the motor impairment and ataxia observed in HD. Cerebellar Purkinje cells (PCs) are responsible for the proper cerebellar pathways functioning and motor control. Recent studies on mouse models of HD have shown that the abnormality of the biochemical functions of PCs are observed in HD, suggesting the contribution of PC dysfunction and death to the impaired movement coordination observed in HD. To investigate ataxic symptoms in HD we performed a series of experiments with the yeast artificial chromosome transgenic mouse model of HD (YAC128). Using extracellular single-unit recording method we found that the portion of the cerebellar PCs with bursting and irregular patterns of spontaneous activity drastically rises in aged YAC128 HD mice when compared with wild type littermates. Previous studies demonstrated that SK channels are responsible for the cerebellar PC pacemaker activity and that positive modulation of SK channel activity exerted beneficial effects in different ataxic mouse models. Here we studied effects of the SK channels modulator chlorzoxazone (CHZ) on the motor behavior of YAC128 HD mice and also on the electrophysiological activity and neuroanatomy of the cerebellar PCs from these mice. We determined that the long-term intraperitoneal injections of CHZ alleviated the progressive impairment in the firing pattern of YAC128 PCs. We also demonstrated that treatment with CHZ rescued age-dependent motor incoordination and improved the cerebellar morphology in YAC128 mice. We propose that abnormal changes in the PC firing patterns might be a one of the possible causes of ataxic symptoms in HD and in other polyglutamine disorders and that the pharmacological activation of SK channels may serve as a potential way to improve the activity of cerebellar PCs and relieve the ataxic phenotype in HD patients.

## Introduction

Huntington’s disease (HD) represents an autosomal dominant neurodegenerative disease that initially presents itself in patients aged 35–50 years old that unrelentingly leads to the patient’s death within 15–20 years after the initial manifestation. HD is characterized by the motor function impairment, including chorea, and also by the psychiatric disturbances associated with a progressive dementia (Vonsattel and DiFiglia, 1998). The degeneration of the GABAergic medium spiny neurons (MSNs) in the striatum is primarily observed in HD (Vonsattel et al., 1985; Vonsattel and DiFiglia, 1998; Eidelberg and Surmeier, 2011). The disease-causing gene is *HTT* with extra CAG repeats coding for the huntingtin protein (htt) with an excessive polyglutamine (polyQ) expansion in the N-terminal domain (The huntington’s disease collaborative research group, 1993). The htt has more than 35 Q residues in HD. The amount of CAG triplets is inversely proportional to the age of the disease manifestation (Langbehn et al., 2004). The htt protein is ubiquitously translated in the brain and other areas and the level of its translation in the striatum is similar to the other areas of the brain and body (Li et al., 1993). Htt has an important role in the ontogenesis and the *HTT* deletion leads to the prenatal death in mice (Nasir et al., 1995). The htt is implicated in the signal transduction (MacDonald, 2003), vesicular transport, cell division, ciliogenesis, autophagy, transcription, and cell survival. Htt also has many other functions, but the pathological pathway of the mutant htt still remains unclear (Saudou and Humbert, 2016).

Many transgenic mouse models were created to study HD pathogenesis (Heng et al., 2008; Stricker-Shaver et al., 2018). The early HD symptoms are most likely caused by the disruption of the synaptic connection between cortex and striatum (Milnerwood and Raymond, 2010). Thus, the majority of HD research is focused on the study of the synaptic transmission between cortical neurons and striatal MSNs (Miller and Bezprozvanny, 2010; Milnerwood and Raymond, 2010; Wu et al., 2016). However, it has been known for quite some time that the cerebellar atrophy is also observed in HD (Rodda, 1981). Studies on HD patients have shown that the cerebellar degeneration occurs during the early stages of the disease independently from the striatal atrophy (Rub et al., 2013; Wolf et al., 2015). The studies on R6/2 transgenic murine model of HD have revealed a reduction in the expression levels of the calcium-binding proteins and cerebellar Purkinje cells (PCs) markers parvalbumin and calbindin that mostly occurred in the cerebellar PCs layer, PC loss was observed as well, and the decrease in the PC firing rate was detected in presymptomatic R6/2 mice (Dougherty et al., 2012). Similar findings were obtained in the HdhQ200 knock-in murine model of HD (Dougherty et al., 2013). Nevertheless, *in vivo* studies of PCs firing have not been carried out yet in the murine models of HD. To fulfill this gap, during the performed research we studied the physiological features of PCs from the intact cerebella of wild-type (WT) and YAC128 transgenic HD mice using extracellular *in vivo* recording technique.

The cerebellum controls the muscular functions and motor behavior. The cerebellar PCs represent the primary functioning unit in the cerebellum since their axons form the sole way out of the cerebellar cortex to the cerebellar nuclei and other deep brain areas (Egorova and Bezprozvanny, 2019). The cerebellar PCs intrinsically generate spikes at a stable frequency (Llinas and Sugimori, 1980; Nam and Hockberger, 1997; Raman and Bean, 1997, 1999; Womack and Khodakhah, 2002; Smith and Otis, 2003; De Zeeuw et al., 2011). This tonic spike generation of the PCs seems to be critical for the proper transmission of the cortical cerebellar data (Hoebeek et al., 2005; De Zeeuw et al., 2011; Kasumu et al., 2012a). Numerous studies have shown the disturbances in the pacemaking activity of the cerebellar PCs in different murine models of ataxia when compared to the WT PCs of the same age (Walter et al., 2006; Alvina and Khodakhah, 2010b; Shakkottai et al., 2011; Kasumu et al., 2012a, b; Collins et al., 2013; Dell’Orco et al., 2015; Mark et al., 2015; Egorova et al., 2016). These data propose a hypothesis that the initial disease manifestation can be explained by the disturbances in firing patterns of PCs. It has been assumed that the pharmacological correction of the abnormal PC firing might have a therapeutic potential in ataxia (Chopra and Shakkottai, 2014).

The positive modulation of small-conductance calcium-activated potassium (SK) channels activity has been suggested as a way of therapeutic regulation of abnormal PC activity as the PCs pacemaking is regulated by these channels (Womack and Khodakhah, 2003). Indeed, the beneficial effects have been demonstrated with SK channel positive modulators chlorzoxazone (CHZ) and 1-ethyl-2-benzimidazolinone (1-EBIO) that normalized the accuracy of PCs spike generation and improved the motor performance in a murine model of episodic ataxia type 2 (EA2) (Walter et al., 2006; Alvina and Khodakhah, 2010a, b). Exposure to the SK channels activator SKA-31 alleviated the ataxic symptoms in a SCA3 murine model by fixing the impaired PC pacemaking activity and correcting motor performance in SCA3 mice (Shakkottai et al., 2011). Our laboratory have shown that the activator of SK2/3 channels (NS13001) normalized PC pacemaking activity and improved the motor performance and PC morphology in SCA2-58Q transgenic mice (Kasumu et al., 2012a). Riluzole trial was successful in a phase II clinical study in patients with different ataxia types (Ristori et al., 2010) and in a subsequent trial with a greater number of patients with inherited cerebellar ataxia (Romano et al., 2015). However, no studies on the treatment of the ataxic symptoms in HD mice have been previously performed. Here we tested the use of FDA-approved drug CHZ as a novel treatment of ataxic symptoms in HD.

## Materials and Methods

### Mice Breeding and Genotyping

Experimental procedures were carried out with transgenic YAC128 HD mice and their wild type (WT) littermates. YAC128 mice with FVBN/NJ background strain have been received from Jackson Labs (stock number 004938) and bred as previously described (Slow et al., 2003). Briefly, heterozygous male YAC128 mice were crossed with the WT female mice to generate mixed litters. Next, the *HTT* transgene detection was performed via PCR. The volume of one PCR sample was 25 μl. The PCR mix per one sample contained: 2.5 μl 10 × buffer for Taq polymerase, 0.5 μl 10mM dNTP, 1,5 μl 25 mM MgCl_2_, 0.125 μl 20 μM primers (forward and reverse), 0.25 μl Taq polymerase, 2 μl DNA, and 18 μl dH2O. The nucleic acid sequence of the forward primer was: 5′-CCGCTCAGGTTCTGCTTTTA-3′. The nucleic acid sequence of the reverse primer was: 5′-TGGAAGGACTTGAGGGACTC-3′. The PCR product had 170 bp. The laboratory mice were housed in groups of two to six in the vivarium. The temperature was kept 22–24°C with the 12 daylight hour cycle. The animals were provided with standard food and water *ad libitum*. The animal study was reviewed and approved by the Bioethics Committee of the Peter the Great St. Petersburg Polytechnic University at St. Petersburg, Russia and followed the principles of European convention (Strasbourg, 1986)^1^ and the Declaration of International medical association about humane treatment of animals (Helsinki, 1996)^2^.

### Extracellular Single-Unit Recordings *in vivo*

The *in vivo* recording technique of PCs spike generation was adjusted from a reported study (Gao et al., 2012) and was carried out as previously described (Egorova et al., 2016, 2018). Briefly, the animals were sedated firstly with 1,200 mg/kg urethane. Then in 40 min this concentration was increased to 1,800 mg/kg. The cerebellar PCs spike generation was observed from 1 to 6 h after the last injection with anesthetic. After sedative effects were attained, the mice were fixed using a stereotaxic apparatus (RWD Life Science, CA, United States). The body heat of the animals was kept at 37°C via a feedback-controlled heating pad (Harvard Apparatus, MA, United States). Further, the scalp under cerebellum was removed and a burr hole was bored into the skull surface under lambdoidal suture. Extracellular recordings of PCs spike generation were recorded from IV – V cerebellar lobules using borosilicate glass pipettes (1.5 mm outer diameter, 0.86 mm inner diameter, Sutter Instruments, CA, United States) filled with 2.5 M NaCl and resistance of 3–10 MΩ. The glass electrodes were forwarded into the brain using a one-axis oil hydraulic micromanipulator (Narishige Group, Japan) and the firing activity was steadily traced. To set a baseline activity, PC spike generation was observed for at least 5–10 min before the recording. The PC activity was detected by means of the complex spike (CS) generation as previously described (Egorova et al., 2018). The production of complex spikes happens due to the climbing fiber excitation leading to the occurrence of calcium-mediated action potentials in the dendrites, and simple spikes are generated due to the synaptic activation by the parallel fibers also known as the axons of the granule cells (Raman and Bean, 1999). The AC/DC Differential Amplifier (A-M Systems. Inc., WA, United States) was used to enhance the registered electrophysiological signals, which were then filtered (100 Hz high pass and 10 kHz low pass filters), digitized via analog-to-digital converter NI PCI-6221 (National Instruments, TX, United States) and were stored for off-line computer analysis. For data collection and analysis, the Bioactivity Recorder v. 5.9 software was applied. Further analysis was performed via Clampfit v10.3.1.5 and Origin software.

Spontaneous activity of cerebellar PCs was recorded for at least 30 s after at least 5 min observation from each cell. During each recording, PC spike generation was identified as tonic, irregular, or bursting as previously described (Kasumu et al., 2012b; Egorova et al., 2016). A PC was classified as generating tonic activity if it has exhibited repetitive tonic spikes occurrence with a constant frequency. A PC was classified as bursting if it was detected that it had more than 5 per cent of the interspike intervals (ISIs) that outlay outside of 3 SDs from the mean of all ISIs in that PC (Kasumu et al., 2012b). The PC spike generation was characterized as irregular if patterns of activity with different firing frequency were clearly observed, the probability density of ISI had shape that couldn’t be related to any known statistical distribution, and the coefficient of variation (CV) ISI was above 0.5 (Egorova et al., 2016). The fraction of cerebellar PCs with tonic activity for each group at 12 months of age was estimated and presented as mean percentage of all observed PCs ± SE. Cerebellar PCs with tonic activity were analyzed further for the type of spike generation pattern and CS shape properties as previously described (Egorova et al., 2018).

Thus, to evaluate the bioelectrical features of PC spike generation patterns, the mean values of simple spike frequency, complex spike frequency, and post-CS pause were assessed. The post-CS pause was determined as time interval from the end of CS to the first SS generation as previously described (Egorova et al., 2018). To define the complex spike shape, mean values of complex spike duration, spikelet frequency, and spikelet number were assessed as previously described (Egorova et al., 2018). The experimental results on the PC spike generation pattern and CS shape in WT and YAC128 PCs were plotted as mean ± SE. The coefficient of variation of ISI (CV ISI) was estimated to assess the firing variability. The CV ISI was counted as the SD divided by the mean ISI in a 30 s observed time period for each PC.

### Immunostaining

Mice were transcardially perfused with phosphate-buffered saline (PBS, pH 5.4) until the output fluid cleared up, after that 4% paraformaldehyde (PFA) was applied. The brain was dissected and postfixed in 4% PFA overnight at 4°C. Later brain was dehydrated in 30% sucrose for ~48 h before cerebellum dissection and slicing. Subsequently, 50-um-thick sagittal slices were made with a NVSLM1 motorized advance vibroslice (World Precision Instruments). Further, cerebellar slices were rinsed two times in PBS and then exposed to the acetone at -20°C for 30 min to increase the tissue antigenicity (Mansouri et al., 2007), then rinsed once in PBS and incubated overnight in 0.5% Triton X-100 in PBS at 4°C (Mansouri et al., 2007). Next, slices were treated with a blocking solution [0.5% Triton X-100 and 2% bovine serum albumin (BSA) in PBS] for 1 h at a room temperature, and then were incubated for 48–72 h at 4°C in blocking solution containing primary antibodies, mouse monoclonal anti-calbindin-D-28K antibody (C9848, Sigma-Aldrich) at 1:900 and rabbit polyclonal VGLUT2 antibody (42-7800, Invitrogen) at 1:300. Following four washes in PBS, slices were treated with secondary antibodies in blocking solution for 48–74 h at 4°C. Alexa Fluor 594 goat anti-mouse (A11005, Invitrogen) and Alexa Fluor 488 goat anti-rabbit (A11008, Life technologies) were used at 1:500. Finally, slices were rinsed four times in PBS and mounted onto slides with ProLong Gold antifade reagent (P36934, Invitrogen) and further visualized by a confocal microscope (ThorLabs).

### Assessment of Molecular Layer Thickness and CF Innervation Range

To analyze the thickness of the molecular layer and the CF innervation range along the PC dendrite, 20X images were handled via a public domain Java image processing program ImageJ (NIH Image). The thickness of the molecular layer was assessed in the primary fissure and was estimated as the interval from the bottom of the PC body to the tip of the dendritic arbor next to the pial surface, as previously described (Barnes et al., 2011). The longest distance along which CFs terminals ascended on the PC dendritic arbor from the bottom of the PC body was estimated relative to the molecular layer thickness (Barnes et al., 2011). Nine measurements per section from three slices taken from three different mice per experimental group were taken for both parameters, resulting in a comparison of 81 individual measurements from three mice per each experimental group.

### Drug Delivery in Mice

Female mice from each litter were genotyped, weight-matched, and divided into two WT and two transgenic YAC128 groups, with each group including 9–12 mice. CHZ was intraperitoneally (i.p.) injected into mice twice a week in the 30 mg/kg concentration, diluted in 5% DMSO in PBS. The control groups of mice were given vehicle i.p. injections with the same periodicity. The injections were done to mice from 2 to 11 months of age. Between 11 and 12 months of age, all animals were injected with a control solution only (washout).

In accordance with our previous studies of the cerebellar PC firing in SCA2-58Q mice (Kasumu et al., 2012b; Egorova et al., 2016, 2018), the YAC128 HD mice and their WT littermates at the age of 6 and 9 months did not have any significant differences between the spontaneous activity of cerebellar PCs between male and female mice of each genotype. However, we chose to set up the CHZ trial on female mice because the male mice are becoming much more aggressive with age compared to female mice, independently on the mice genotype (Van Loo et al., 2003).

### Motor Coordination Assessments in Mice

The motor coordination assay with YAC128 HD mice was set up as previously described (Liu et al., 2009; Chen et al., 2011). The beam-walk test was carried out via a home-made testing setup featuring a beam of 1.5 m suspended 0.5 m above the ground. The 18, 12, and 8 mm round wood beams were used in the performed tests. At each time point, the animals were practicing on the beams for 3 consecutive days (three training sessions per day) to traverse the beam to the enclosed box. Once the stable baseline of performance was achieved, the animals’ results were recorded in three consecutive trials on 18, 12, and 8 mm round wood beams, each time measuring from the widest to the narrowest beam. The latency to traverse the middle 1 m of each beam and the number of times the hind paws slipped off each beam were assessed for each trial. For those animals with “crawling behavior,” their every crawling step was registered as one foot slip. The motor coordination assessment was estimated based on the average scores of the three trials for each beam for each mouse.

## Statistical Analysis

The statistically significance of the difference between the experimental groups was estimated via one-way ANOVA/Bonferroni post-test.

## Results

### Extracellular Single-Unit Recordings Showed Disturbances in the Spontaneous Spike Generation of the Cerebellar PCs in Aging YAC128 HD Mice

**FIGURE 1:**
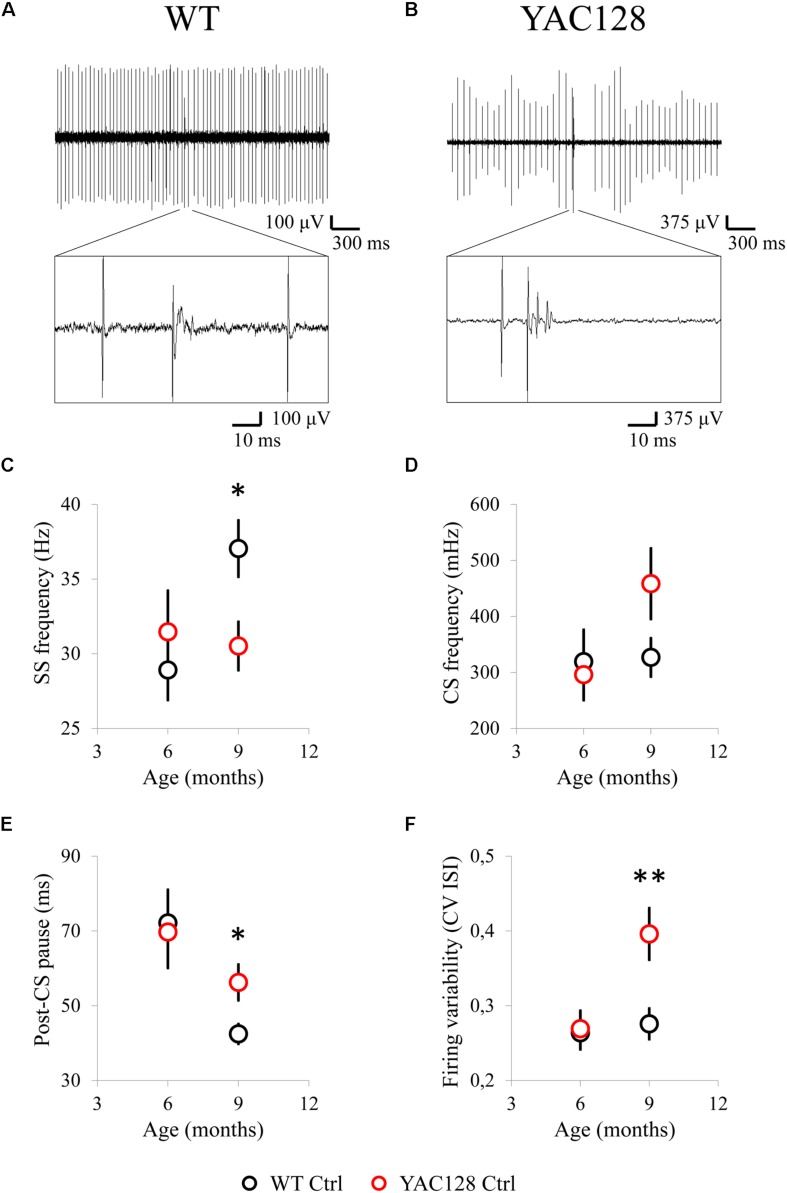
Spontaneous activity of cerebellar PCs in 6- and 9-month-old WT and YAC128 HD mice. **(A,B)** Examples of PC firing activity in 9-month-old WT **(A)** and YAC128 **(B)** mice. Recording traces are 3 s in duration with enlarged 100-ms fragment containing CS is shown. **(C)** The average SS firing frequency of PCs for 6- and 9-month-old WT (*n* = 23 and 34 cells; *m* = 10 and 7 mice) and YAC128 mice (*n* = 24 and 31 cells; *m* = 6 and 7 mice) is shown as mean ± SE. *p < 0.05. **(D)** The average CS firing frequency of PCs for 6- and 9-month-old WT (*n* = 23 and 34 cells; *m* = 10 and 7 mice) and YAC128 mice (*n* = 24 and 31 cells; *m* = 6 and 7 mice) is shown as mean ± SE. No statistically significant difference was observed. **(E)** The average post-CS pause in PC discharge for 6- and 9-month-old WT (*n* = 23 and 34 cells; *m* = 10 and 7 mice) and YAC128 mice (*n* = 24 and 31 cells; *m* = 6 and 7 mice) is shown as mean ± SE. *p < 0.05. **(F)** The CV ISI of tonically firing PCs was analyzed. Tonically firing PCs in 9-month-old HD mice fired more irregularly. **p < 0.01.

In preceding studies with acute cerebellar slices it was shown that PC firing rate is reduced in presymptomatic R6/2 mice at the age of 4 weeks (Dougherty et al., 2012) as well as in 50 week old homozygous HdhQ200 mice (Dougherty et al., 2013). Here we carried out a group of *in vivo* extracellular recordings of PC spike generation in WT (Figure 1A) and YAC128 (Figure 1B) mice at the age of 6 and 9 months. We discovered that the mean PC simple spike (SS) firing frequency (FF) was similar in 6-month-old YAC128 mice and their WT littermates, but was significantly reduced in 9-month-old YAC128 HD mice (Figure 1C). Thus, the average SS FF for 6-month-old WT mice was 28.9 ± 2.0 Hz (*n* = 23 cells, *m* = 10 mice) and for 6-month-old YAC128 mice was 31.5 ± 2.7 Hz (*n* = 24, *m* = 6, ns) (Figure 1C). The average SS FF for 9-month-old WT mice was 37.0 ± 1.9 Hz (*n* = 34, *m* = 7) and for 9-month-old YAC128 mice was 30.5 ± 1.6 Hz (*n* = 31, *m* = 7, ^∗^ *p* < 0.05) (Figure 1C). We observed no statistically significant difference in complex spike (CS) FF in 6- and 9-month-old WT and YAC128 mice (Figure 1D). Thus, the average CS FF for 6-month-old WT mice was 319 ± 57 mHz (*n* = 23 cells, *m* = 10 mice) and for 6-month-old YAC128 mice was 296 ± 45 mHz (*n* = 24, *m* = 6, ns) (Figure 1D). The average CS FF for 9-month-old WT mice was 327 ± 34 mHz (*n* = 34, *m* = 7) and for 9-month-old YAC128 mice was 458 ± 63 mHz (*n* = 31, *m* = 7, ns) (Figure 1D). We further discovered that the average post-CS pause (the depression of SS) in the PC discharge was similar in 6-month-old YAC128 mice and their WT littermates, but was significantly increased in 9-month-old YAC128 HD mice (Figure 1E). Thus, the average post-CS pause for 6-month-old WT mice was 72.1 ± 8.9 ms (*n* = 23 cells, *m* = 10 mice) and for 6-month-old YAC128 mice was 69.7 ± 9.6 ms (*n* = 24, *m* = 6, ns) (Figure 1E). The average post-CS pause for 9-month-old WT mice was 42.5 ± 2.6 ms (*n* = 34, *m* = 7) and for 9-month-old YAC128 mice was 56.2 ± 4.7 ms (*n* = 31, *m* = 7, ^∗^ *p* < 0.05) (Figure 1E). Next, we determined the average CV ISI for the all PCs with tonic activity in all studied experimental groups (Figure 1F). We observed that the firing variability of ISIs was alike for WT and YAC128 mice at 6 months of age, but was noticeably higher in YAC128 mice at 9 months of age compared to their WT littermates (Figure 1F). Thus, the average CV ISI for 6-month-old WT mice was 0.26 ± 0.02 (*n* = 23 cells, *m* = 10 mice) and for 6-month-old YAC128 mice was 0.27 ± 0.02 (*n* = 24, *m* = 6, ns) (Figure 1F). The average CV ISI for 9-month-old WT mice was 0.28 ± 0.02 (*n* = 34, *m* = 7) and for 9-month-old YAC128 mice was 0.40 ± 0.03 ms (*n* = 31, *m* = 7, ^∗∗^ *p* < 0.01) (Figure 1F). No statistically significant difference was found in CS shape properties for 6- and 9-month-old YAC128 mice and their WT littermates (data not shown).

Obtained *in vivo* recordings suggest that cerebellar PCs fire less frequently in aging YAC128 HD mice compared to their WT littermates and this observation is coincident with the preceding results obtained in the experiments with cerebellar slices from WT and HD mice (Dougherty et al., 2012, 2013). Interestingly, in the research with acute cerebellar slices we earlier demonstrated that tonically firing PCs in the murine model of spinocerebellar ataxia type 2 (SCA2-58Q mice) also exert decreased spike generation compared to their WT littermates (Kasumu et al., 2012a, b). Cerebellar PCs from SCA2-127Q mice (Hansen et al., 2013) and from the fast-activating/deactivating voltage-gated potassium channel Kv3.3 mutant mice (Hurlock et al., 2008) revealed the same decrease in tonic spike generation during the performed cerebellar slice recordings. Dominant-negative mutations in Kv3.3 are observed in SCA13 patients.

### CHZ Improves the Spike Generation of Cerebellar PCs From Aged YAC128 HD Mice

**FIGURE 2:**
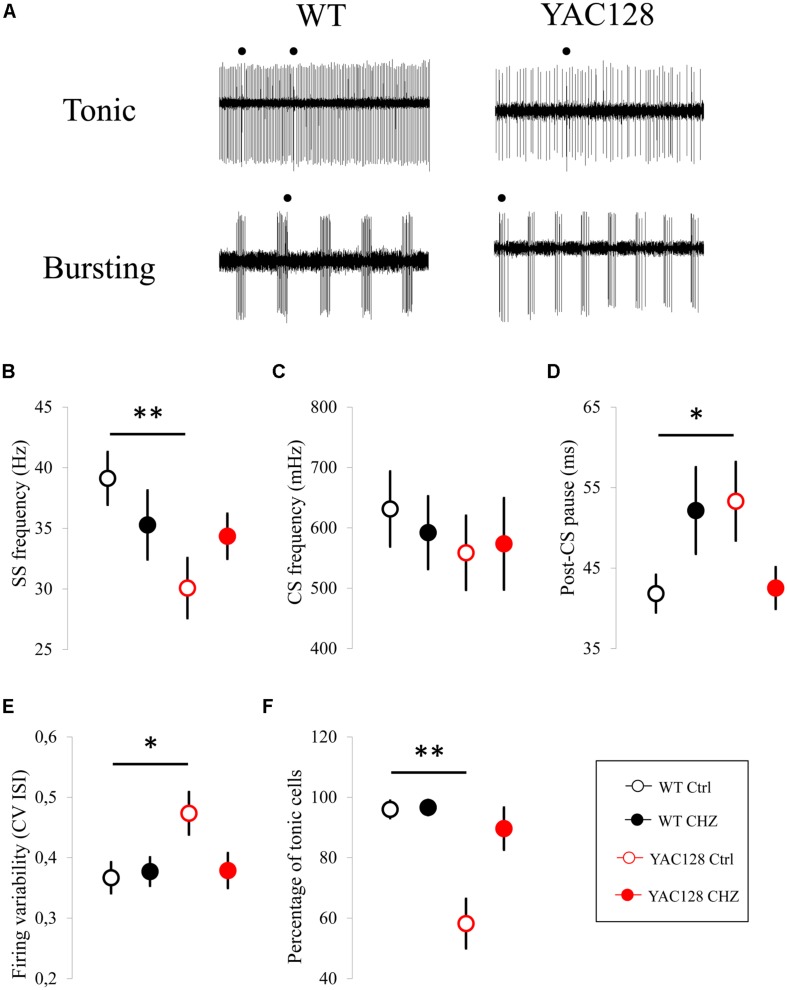
Long-term injections of 30 mg/kg CHZ alleviate the age-dependent dysfunction in the firing pattern of 12-month-old HD PCs. **(A)** Examples of tonic and bursting PC activity in 12-month-old WT and YAC128 mice. Recording traces 3 s in duration are shown. Complex spikes are marked by filled circles. **(B)** The average SS firing frequency was significantly less in 12-month-old YAC128 mice compared to WT mice and was recovered after treatment with CHZ. **p < 0.01. **(C)** No statistically significant difference was observed in CS firing frequency between all the experimental groups. **(D)** The average post-CS pause in PC discharge was significantly higher in 12-month-old YAC128 mice compared to WT mice and was recovered after treatment with CHZ. *p < 0.05. **(E,F)** CHZ injections recover the precision of the PC pacemaker activity in 12-month-old YAC128 HD mice. At 12 months of age, the systematic CHZ injections decreased the firing variability **(E)** and increased the proportion of tonically firing cells **(F)** in YAC128 HD mice. *p < 0.05, **p < 0.01. For all the measured properties number of tonic cells (n) and number of animals (m) were following: *n* = 51 cells for WT mice in the control group (WT Ctrl), *m* = 7 WT Ctrl mice; *n* = 51 cells for WT mice injected with CHZ, *m* = 8 WT CHZ mice; *n* = 40 cells for YAC128 mice in the control group (YAC128 Ctrl), *m* = 11 YAC128 Ctrl mice; *n* = 34 cells for YAC128 mice injected with CHZ, *m* = 9 YAC128 CHZ mice.

**FIGURE 3:**
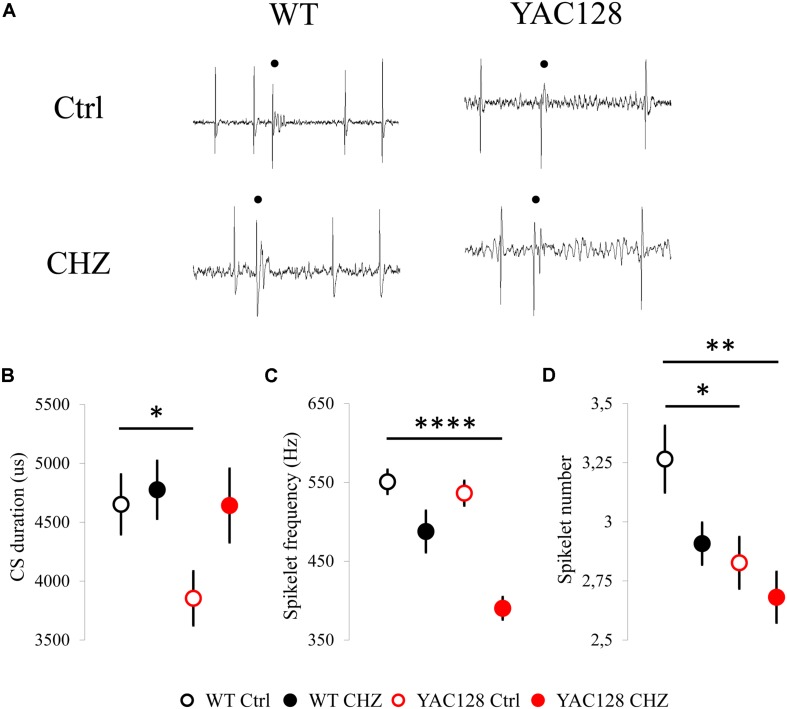
Changes in CS shape induced by long-term CHZ injections in 12-month-old WT and YAC128 HD mice. **(A)** Examples of complex spikes generated by cerebellar PCs from 12-month-old WT and YAC128 mice in control groups and after long-term CHZ injections. Complex spikes are marked by filled circles. **(B)** The average CS duration was significantly less in 12-month-old YAC128 mice compared to WT mice and was recovered after treatment with CHZ. *p < 0.05. **(C)** After CHZ treatment the significant decrease in spikelet frequency of the CS from YAC128 PCs was observed. ****p < 0.0001. **(D)** The spikelet number of the CS from YAC128 PCs was significantly less in both YAC128 control and CHZ groups compared to WT PCs. *p < 0.05, **p < 0.01. The number of the analyzed tonic cells (n) and number of animals (m) were indicated in Figure 2 legend.

We earlier demonstrated that SK activators can improve abnormal spike generation of ataxic PCs in cerebellar slice recordings and alleviate the motor coordination in aging SCA2-58Q ataxic mice (Kasumu et al., 2012a). We also have shown that activators of SK channels affect similarly the spike generation in SCA2 mice *in vivo* via extracellular single-unit registration of PCs firing before and after i.p. injections with 60 mg/kg of a positive SK channel modulator chlorzoxazone (CHZ) using 6 months old SCA2-58Q mice (Egorova et al., 2016). Here, in the following studies we applied the resembling experimental design to assess the physiological impact of CHZ in WT and YAC128 12-month-old mice (Figures 2, 3). The advantage of CHZ is that this compound has been studied in many preclinical trials as possible therapeutic agent for episodic ataxia type 2 (EA2) (Alvina and Khodakhah, 2010a), cystic fibrosis, hypertension (Syme et al., 2000) and also for excessive alcohol intake (Hopf et al., 2011) and some other disorders. Though molecular mechanisms of CHZ actions are still not well defined, it had been conducted a few series of clinical trials with CHZ as a drug, the last one completed study was on placebo-controlled cross over trial of CHZ intake under the condition of alcohol abuse, which revealed one more time that CHZ therapy can be used in clinic (NTC number of the study: NCT01342341, verification date May 2014).

In the next series of studies the following experimental groups were examined: WT mice in the control group injected with a vehicle (WT Ctrl), WT mice injected with CHZ (WT CHZ), YAC128 mice in the control group (YAC128 Ctrl), and YAC128 mice injected with CHZ (YAC128 CHZ). All mice were at the age of 12 months. As previously, in each experiment, PC activity was classified as tonic, irregular, and bursting as previously described (Kasumu et al., 2012b; Egorova et al., 2016). Both tonic and bursting cells (Figure 2A) were observed in 12-month-old WT and YAC128 mice. The PC properties were evaluated for tonic cells and averaged for all different mice from the same age and genotype. In our studies the long-term i.p. delivery of 30 mg/kg CHZ resulted in the recovery of the SS FF in 12-month-old YAC128 HD mice (Figure 2B). Thus, the average SS FF for WT Ctrl mice was 39.1 ± 2.2 Hz (*n* = 51 cells, *m* = 7 mice), for WT CHZ mice was 35.3 ± 2.8 Hz (*n* = 51, *m* = 8, ns), for YAC128 Ctrl mice was 30.1 ± 2.5 Hz (*n* = 40, *m* = 11, ^∗∗^*p* < 0.01), and for YAC128 CHZ mice was 34.3 ± 1.9 Hz (*n* = 34, *m* = 9, ns) (Figure 2B). Similar to 6- and 9-month-old experimental groups, we did not detect any statistically significant difference in the average CS FF values between the studied groups (Figure 1C). Thus, the average CS FF for WT Ctrl mice was 631 ± 62 mHz (*n* = 51 cells, *m* = 7 mice), for WT CHZ mice was 592 ± 60 mHz (*n* = 51, *m* = 8, ns), for YAC128 Ctrl mice was 559 ± 61 mHz (*n* = 40, *m* = 11, ns), and for YAC128 CHZ mice was 574 ± 75 mHz (*n* = 34, *m* = 9, ns) (Figure 2C). We further observed that long-term CHZ injections recover the average mean of the post-CS pause in the PC discharge (Figure 2D). Thus, the average post-CS pause for WT Ctrl mice was 41.8 ± 2.3 ms (*n* = 51, *m* = 7), for WT CHZ mice was 52.2 ± 5.4 ms (*n* = 51, *m* = 8, ns), for YAC128 Ctrl mice was 53.3 ± 4.9 ms (*n* = 40, *m* = 11, ^∗^ *p* < 0.05), and for YAC128 CHZ mice was 42.5 ± 2.6 ms (*n* = 34, *m* = 9, ns) (Figure 2D). We also discovered that YAC128 CHZ PCs fired as regularly as WT Ctrl PCs (Figure 2E). Thus, the average CV ISI for WT Ctrl mice was 0.37 ± 0.03 (*n* = 51, *m* = 7), for WT CHZ mice was 0.38 ± 0.02 (*n* = 51, *m* = 8, ns), for YAC128 Ctrl mice was 0.47 ± 0.04 (*n* = 40, *m* = 11, ^∗^*p* < 0.05), and for YAC128 CHZ mice was 0.38 ± 0.03 (*n* = 34, *m* = 9, ns) (Figure 2E). And, finally, long-term CHZ administration significantly increased the proportion of tonically firing cells in 12-month-old YAC128 HD mice (Figure 1F). Thus, the average percentage of tonic cells for WT Ctrl mice was 96 ± 3% (*m* = 7), for WT CHZ mice was 97 ± 3% (*m* = 8, ns), for YAC128 Ctrl mice was 58 ± 8% (*m* = 11, ^∗∗^*p* < 0.01), and for YAC128 CHZ mice was 90 ± 7% (*m* = 9, ns) (Figure 2F).

We further evaluated the effect of long-term CHZ injections on the CS shape in the same experimental groups (Figure 3A). We realized that CHZ administration led to the recovery of the average CS duration in 12-month-old YAC128 mice (Figure 3B). Thus, the average CS duration for WT Ctrl mice was 4,652 ± 254 us (*n* = 51, *m* = 7), for WT CHZ mice was 4,776 ± 246 us (*n* = 51, *m* = 8, ns), for YAC128 Ctrl mice was 3,854 ± 229 (*n* = 40, *m* = 11, ^∗^*p* < 0.05), and for YAC128 CHZ mice was 4,643 ± 313 (*n* = 34, *m* = 9, ns) (Figure 3B). Interestingly, while the spikelet frequency was similar between WT and YAC128 control groups, the CHZ treatment led to the significant decrease in the spikelet frequency in YAC128 PCs (Figure 3C). Thus, the average spikelet frequency for WT Ctrl mice was 551 ± 15 Hz (*n* = 51, *m* = 7), for WT CHZ mice was 488 ± 26 Hz (*n* = 51, *m* = 8, ns), for YAC128 Ctrl mice was 536 ± 16 (*n* = 40, *m* = 11, ns), and for YAC128 CHZ mice was 390 ± 14 (*n* = 34, *m* = 9, ^****^ *p* < 0.0001) (Figure 3C). Furthermore, the spikelet number was significantly less in YAC128 Ctrl PCs compared to WT Ctrl PCs and CHZ treatment had no effect on this parameter (Figure 3D). Thus, the average spikelet number for WT Ctrl mice was 3.27 ± 0.14 (*n* = 51, *m* = 7), for WT CHZ mice was 2.91 ± 0.09 Hz (*n* = 51, *m* = 8, ns), for YAC128 Ctrl mice was 2.83 ± 0.11 (*n* = 40, *m* = 11, ^∗^ *p* < 0.05), and for YAC128 CHZ mice was 2.68 ± 0.11 (*n* = 34, *m* = 9, ^∗∗^ *p* < 0.01) (Figure 3D).

### Long-Term i.p. Injections of CHZ Recover the CF Terminal Localization in YAC128 HD Mice

**FIGURE 4:**
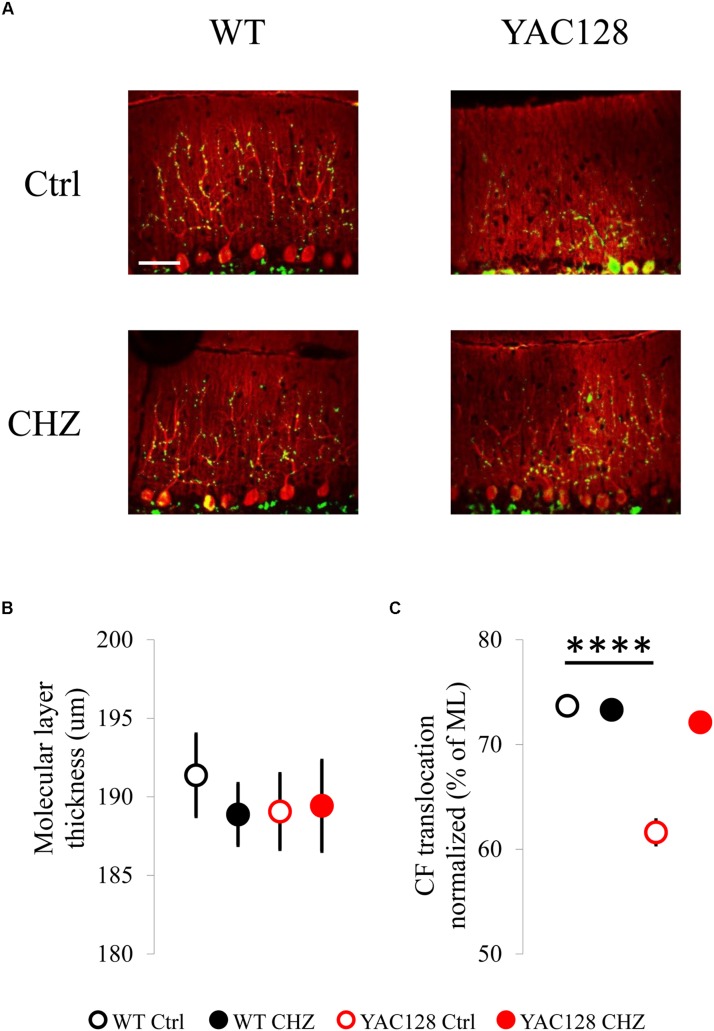
Long-term injections of 30 mg/kg CHZ improved the CF terminal localization in 12-month-old YAC128 HD mice. **(A)** Immunofluorescently-labeled sagittal cerebellar sections show calbindin-positive PCs (red) and VGLUT2-positive CF terminals (green). Images were taken at 20X. Scale bar, 50 μm. **(B)** Molecular layer thickness at the primary fissure was similar in WT and YAC128 mice in control and after the CHZ treatment. **(C)** CF terminal translocation measured as a percent of total molecular layer thickness was significantly less in YAC128 mice compared to WT mice and was recovered after long-term CHZ injections. ****p < 0.0001. For each experimental group number of animals *m* = 3, number of measurements *n* = 81. All results are shown as mean ± SE.

Previous experiments on R6/2 and HdhQ200 HD mice have demonstrated the reduction in the expression and immunoreactivity for calbindin in these mice (Dougherty et al., 2012, 2013). Here, we performed anti-calbindin immunostaining technique to evaluate the molecular layer (ML) thickness of mice cerebella (Figure 4A). To quantify morphological changes in the cerebellum of 12-month-old YAC128 HD mice, we measured the ML thickness (Figure 4B). We discovered that the ML thickness was similar in all groups tested, although it was insignificantly higher in WT Ctrl mice (191 ± 3 μm) than in YAC128 Ctrl mice (189 ± 2 μm). CHZ treatment did not affect this parameter in WT CHZ (189 ± 2 μm) and YAC128 CHZ (189 ± 3 μm) groups (Figure 4B). We also performed anti-VGlut2 immunostaining to evaluate the climbing fiber (CF) translocation along the cerebellar PC dendritic arbor (Figure 4A). We found that the CF translocation in YAC128 Ctrl mice (62 ± 1% of the thickness of the molecular layer) was significantly lower than in WT Ctrl group (74 ± 1%) and recovered after CHZ treatment in YAC128 CHZ mice (72 ± 1%), while CHZ administration did not affect WT CHZ mice (73 ± 1%), for each genotype *n* = 81 measurements, *m* = 3 mice, ^****^p < 0.0001 (Figure 4C).

### CHZ Rescues the Motor Coordination Impairments in YAC128 HD Mice

The extracellular recordings of the cerebellar PC activity from YAC128 HD mice suggested that electrophysiological properties of PCs are disturbed in these mice (Figures 1–3). It is known, that SK channels regulate the cerebellar PCs spontaneous spike generation (Womack and Khodakhah, 2003). Here, we showed that pharmacological activation of SK channels by CHZ recovered simple spike generation in YAC128 mice (Figure 2B), improved the firing regularity of cerebellar PCs (Figures 2E,F), and alleviated the cerebellar morphology in 12-month-old YAC128 HD mice (Figure 4). These results indicated that the positive modulation of SK channels activity may have a potential therapeutic impact to cure the ataxic symptoms in HD. To test this hypothesis *in vivo*, we set a CHZ trial in YAC128 HD mice.

The design of CHZ trial was following. Starting at 2 months of age, WT and YAC128 mice were systematically intraperitoneally (i.p.) injected twice a week with 30 mg/kg CHZ diluted in 5% DMSO in PBS, animals from the control groups were injected with a vehicle solution. The concentration of CHZ was chosen according to the previous trials in rodents (Baek et al., 2006). The i.p. injections were chosen to be sure that all the mice actually get the equal drug treatment and also because the pharmacokinetics of substances i.p. administered are more similar to those seen after oral administration (Turner et al., 2011). The CHZ administration was ended at the point of 11 months with a subsequent 1 month washout period, which was initiated to study the difference between long-term and acute influence of CHZ on the motor coordination of the experimental animals.

**FIGURE 5:**
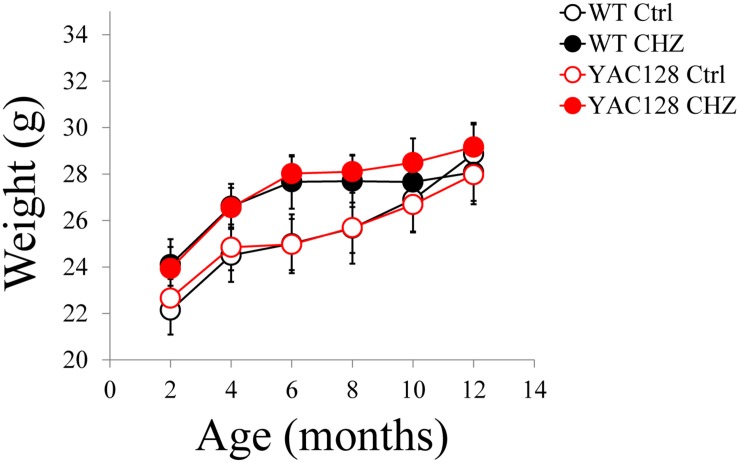
Average body weight for WT and YAC128 mice in control and CHZ treatment groups was calculated and plotted as mean ± SE. Number of mice, *m* = 9 for WT Ctrl mice, *m* = 11 for YAC128 Ctrl mice, *m* = 12 for WT CHZ mice, and *m* = 12 for YAC128 CHZ mice. No statistically significant difference was observed.

**FIGURE 6:**
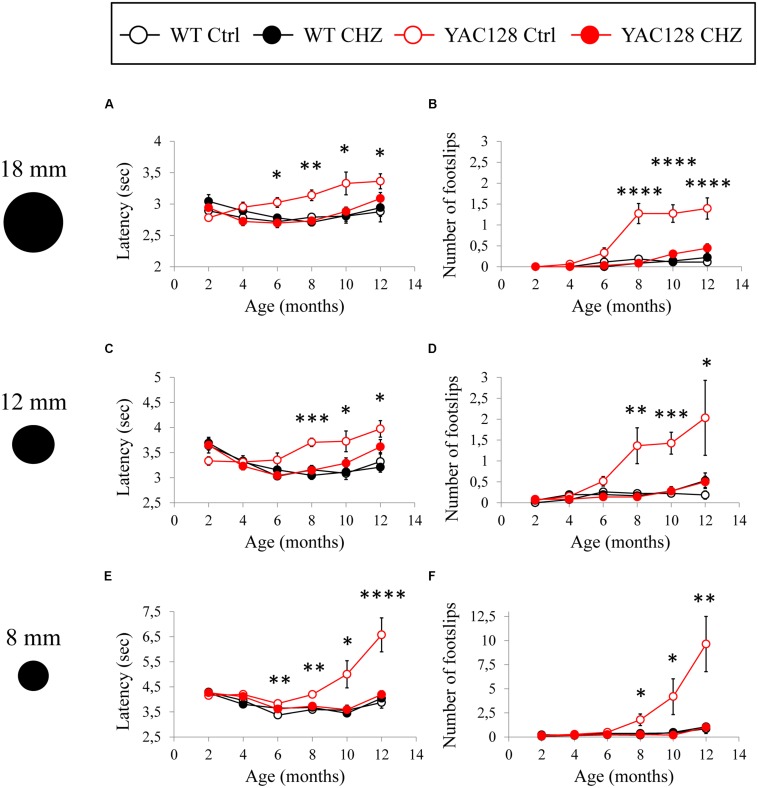
CHZ treatment improves performance of YAC128 mice in the beam-walk test. The average mean latency as the mice traverse the 1 m length of the 18 mm **(A)**, 12 mm **(C)**, and 8 mm **(E)** beam is plotted for the 2, 4, 6, 8, 10, and 12-month-old WT and YAC128 mice in control and CHZ treatment groups as mean ± SE. Average number of foot slips as the mice traverse the 1 m length of the 18 mm **(B)**, 12 mm **(D)**, and 8 mm **(F)** beam is plotted for the 2, 4, 6, 8, 10, and 12-month-old WT and YAC128 mice as mean ± SE. *p < 0.05; **p < 0.01; ***p < 0.001; ****p < 0.0001 significant differences between control WT group and control YAC128 group.

The motor performance of the four experimental groups was analyzed every 2 months by the beam-walk assay. We made sure that the average body weight was equal between all the experimental groups of mice at the same age (Figure 5). The beam-walk test was performed on 18 mm, 12 mm, and 8 mm round wood beams. Motor coordination of mice on each beam was assessed every 2 months as the “latency” and the “number of foot slips” as previously described (Liu et al., 2009; Chen et al., 2011). Just before the beginning of CHZ treatment, the basic value was assessed in 2-month-old animals. Beam-walking test discovered the age-dependent disturbances in motor behavior in YAC128 Ctrl mice compared to WT Ctrl mice (Figure 6). Started from 6 months of age, YAC128 Ctrl group revealed a progressive motor disturbance such as extended beam cross latencies and higher number of foot slips when compared with the WT Ctrl group (Figures 6A–F). Considerable differences between WT Ctrl and YAC128 Ctrl mice were detected started from the age of 6 months for the latency on 18 and 8 mm round beams (Figures 6A,E, ^∗^*p* < 0.05, ^∗∗^*p* < 0.01), started from the age of 8 months for the latency on all used beams and all further ages (Figures 6A,C,E, ^∗^*p* < 0.05; ^∗∗^*p* < 0.01; ^∗∗∗^*p* < 0.001; ^****^p < 0.0001), started from the age of 8 months for foot slips on all used beams and all further ages (Figures 6B,D,F, ^∗^*p* < 0.05; ^∗∗^*p* < 0.01; ^∗∗∗^*p* < 0.001; ^****^p < 0.0001).

Long-term CHZ administration had no statistically significant impact on the motor behavior of WT mice (Figure 6). In contrast, long-term i.p. injections with CHZ drastically alleviated the motor coordination of YAC128 HD mice by reducing the latencies and diminishing the number of foot slips (Figure 6). Started from the age of 6 months, the animals in the YAC128 CHZ group exhibited improved performance to cross the beam compared to YAC128 Ctrl mice (Figures 6A–F). The motor behavior of YAC128 CHZ group was alike to the motor behavior of WT Ctrl group on all used beams at all ages examined (Figures 6A–F). The improved motor coordination of YAC128 mice was not due to the instant impact of CHZ, and it was continued after a 1 month CHZ washout (Figures 6A–F).

During beam-walk test performance, some of the older YAC128 Ctrl mice exerted a “crawling behavior,” an abnormal lack of motor skills in mice. These observations were previously reported in aging YAC128 HD mice (Chen et al., 2011) and some ataxic mice such as SCA3–YAC–84Q mice (Chen et al., 2008) and SCA2-58Q mice (Liu et al., 2009). At 8 months of age, one mouse in the YAC128 Ctrl group crawled on the 8 mm round beam. At 10 months of age, the amount of mice in the YAC128 Ctrl group that crawled on 8 mm beam rose to three mice. At 12 months of age, five more mice exerted a crawling behavior in the YAC128 Ctrl group on 8 mm beam and one mouse started to crawl on the 12 mm round beam. Conversely, no crawling mice were detected in the WT Ctrl, WT CHZ, or YAC128 CHZ groups.

## Discussion

### Bioelectrical Features of the Cerebellar PCs in WT and YAC128 HD Mice

The cerebellar pathways play a key role in the motor activity and body coordination. The precise timing of the cerebellar output provides the accuracy and proper movement velocity. The axons of the GABAergic PCs relay the information to the deep cerebellar nuclei (DCN) thus providing the unique output of the cerebellar cortex (Ito, 2002). PCs spontaneously generate simple spikes at a constant frequency in the range 17–150 Hz (Llinas and Sugimori, 1980; Nam and Hockberger, 1997; Raman and Bean, 1997, 1999; Womack and Khodakhah, 2002; Smith and Otis, 2003). This tonic spike generation of cerebellar PCs is required for the proper transmission of the cortical cerebellar information to the DCN and other brain parts related to the motor behavior. Cerebellar PCs are affected in many ataxias (Orr and Zoghbi, 2007; Carlson et al., 2009; Matilla-Duenas et al., 2009), and heavy PC loss is typical for the final stage of the disorder for the majority of patients with ataxia. Nevertheless, the recent studies on the ataxic mouse models have shown that the initial symptoms of ataxia may be observed due to the disturbances in the PCs activity and decline in the spike generation accuracy during the presymptomatic stage, but not the actual PC death. To support this idea, the impairments of tonic PC spike generation have been shown in the experiments with mouse models of EA2 (Walter et al., 2006; Alvina and Khodakhah, 2010a), SCA3 (Shakkottai et al., 2011), SCA2 (Kasumu et al., 2012a; Hansen et al., 2013), SCA1 (Dell’Orco et al., 2015), SCA6 (Mark et al., 2015) and in *tottering* mice with a mutation in P/Q-type voltage-gated calcium channels (Hoebeek et al., 2005).

HD represents a polyQ neurodegenerative disease distinguished by a progressive impairment in a cognitive, motor, and psychiatric activity. Striatal neurons are primarily affected in HD, but the independent atrophy of the cerebellum seems to be another common feature in HD patients (Rub et al., 2013; Wolf et al., 2015). This cerebellar degeneration most likely is involved in motor behavior impairment in HD. The studies on R6/2 transgenic mouse model of HD have demonstrated the early cerebellar PC dysfunction in these mice (Dougherty et al., 2012). Thus, the decrease in the expression level of GABAergic neuronal proteins such as glutamic acid decarboxylase 67 (GAD67) and parvalbumin, and also a typical PC protein calbindin, was determined in 12-month-old R6/2 mice. Furthermore, a significant reduction in PC firing rate was noted before the neuronal PC loss in presymptomatic 4-week-old R6/2 mice (Dougherty et al., 2012). Similar findings were later obtained in the HdhQ200 knock-in mouse model of HD (Dougherty et al., 2013). In this study, the homozygous HdhQ200/Q200 mice exhibited reductions in calbindin mRNA expression, decline in calbindin, parvalbumin, and GAD67 immunoreactivity, significant PC loss, and, finally, a significant decrease in PC firing frequency (Dougherty et al., 2013).

Here, we analyzed *in vivo* the bioelectrical features of individual PCs from YAC128 transgenic HD mice and their WT littermates at different ages. In conformity with the earlier results obtained in the experiments with cerebellar slices from WT and HD mice (Dougherty et al., 2012, 2013), the drastic decrease in the YAC128 PC SS generation was revealed starting at 9 months of age (Figures 1C, 2B). Similar reduction in PC firing activity was previously observed in the studies with slices from murine models of ataxia in SCA2-58Q transgenic mice (Kasumu et al., 2012a, b), in SCA2-127Q mice (Hansen et al., 2013) and in the fast-activating/deactivating voltage-gated potassium channel Kv3.3 mutant SCA13 mice (Hurlock et al., 2008).

Next, we observed the increase in the variability of interspike intervals in YAC128 PCs started in 9-month-old mice (Figures 1F, 2E) when compared with age-matched WT cells. We further showed that the portion of PCs with tonic spike generation is significantly lower in 12-month-old YAC128 HD mice when compared with WT mice at the same age (Figure 2F). Noteworthy, the age of onset of the bioelectrical disturbances in YAC128 PCs at 9 months of age (Figure 1) reflects the beginning of the motor impairment manifestation in YAC128 HD mice in previous (Chen et al., 2011) and present (Figure 6) researches. The results obtained in our experiments (Figures 1F, 2E,F) and in previous analyses of ataxic mouse models (Walter et al., 2006; Alvina and Khodakhah, 2010b; Shakkottai et al., 2011; Kasumu et al., 2012a, b; Collins et al., 2013; Dell’Orco et al., 2015; Mark et al., 2015; Egorova et al., 2016) support the hypothesis that the loss of the precision of PC activity mirrors the impaired condition of these cerebellar neurons and most likely provokes the ataxic symptoms. This observation supports the well-known fact that the proper PC spike generation is crucial for the exact cerebellar timing and correct activity (Walter et al., 2006).

The *in vivo* recording method used in this study allowed us to analyze not only simple spike (SS) generation by PCs, but also less studied complex spike (CS) generation. PCs generate simple spikes when activated by parallel fibers (PFs) projected from the pontocerebellar system, and other circuits such as vestibular nerve and nuclei, the spinal cord, the reticular formation, and a feedback from DCN (Ito, 1984). The PCs fire CSs when the excitation comes through the olivocerebellar circuit (Davie et al., 2008). The inferior olive (IO) forms a unique source of climbing fibers (CFs) to the cerebellar cortex. Each IO neuron has an axonal extension that forms many synaptic connections on the proximal part of a dendritic tree of PCs. PC reacts on the CF discharges with a CS generation (Barmack, 2006). In this study we did not detect any difference in CS frequency between HD and WT animals at any age tested (Figures 1D, 2C). However, we found that the depression of SS after CS generation (post-CS pause) was significantly higher in YAC128 PCs discharge starting at the age of 9 months compared to their WT littermates (Figures 1E, 2D), thus indicating most likely the delay in PF-PC synapse signal transmission in aging HD mice. The decline of PF-PC synaptic connection was previously observed in SCA1 transgenic mice at 40 weeks of age (Barnes et al., 2011).

The CS shape differs a lot among various PCs. The duration of CS, the number and frequency of spikelets are distinct in each cerebellar neuron. This variety implies that CS waveform could reflect the unique features of olivocerebellar activity. The cause for such a diversity is still not clear (Lang et al., 2014). In our study we did not see any significant difference in CS properties between YAC128 and WT animals at the age of 6 and 9 months (data not shown). Nevertheless, we observed the shorter CS duration (Figure 3B) and the decreased spikelet number (Figure 3D) in YAC128 PCs at 12 months of age compared to their WT littermates. Similar reduction in CS duration and spikelet number was observed in 12-month-old WT mice of the same FVB background after the injections with harmaline, an alkaloid that induces high frequency oscillations in the IO neurons (Egorova et al., 2018). Thus, we assume that the observed difference in the CS shape of YAC128 PCs and WT PCs may be explained by the dysfunction of the inferior olive neurons or olivocerebellar tract itself in aged HD mice.

### CHZ as a Potential Treatment of Ataxic Symptoms in HD

The small-conductance calcium-activated potassium channels (SK channels) control the cerebellar PCs spike generation (Womack and Khodakhah, 2003). Constantly increased internal calcium levels decrease the PC spike generation via SK channels (Meera et al., 2016). The pharmacological activation of SK and BK channels exerted some therapeutic effects in ataxia murine models and also some ataxia patients. The mixture of CHZ and baclofen alleviated the firing activity in SCA1 PCs and recovered the hyperexcitability of the PC dendrites in SCA1-82Q mice, and also exerted the beneficial effects on the impaired motor behavior in these mice (Bushart et al., 2018). It was also shown that the SK channel activator 1-EBIO and CHZ improved the abnormal PCs spike generation and alleviated the motor coordination in a murine model of EA2 (Walter et al., 2006; Alvina and Khodakhah, 2010a, b). Treatment with SKA-31, a riluzole analog optimized for the SK channels activation, alleviated the ataxic symptoms in a mouse model of SCA3 by restoring the impaired PC spike generation and by refining the motor performance in SCA3 mice (Shakkottai et al., 2011). The compound NS13001 that activates SK channels type 2 and 3 recovered the PC spike generation and alleviated the motor behavior and PC morphology in SCA2 mice (Kasumu et al., 2012a). Thus, the cerebellar slice perfusion with NS13001 restored the disturbed bursting activity into the normal tonic spike generation, and also resulted in the drastic alleviation in a motor behavior (Kasumu et al., 2012a). i.p. injections with CHZ converted bursting PCs to tonic spike generation state in aging SCA2-58Q animals (Egorova et al., 2016). Another chemical compound riluzole attracts and allosterically activates the SK2 channel molecules (Cho et al., 2018). A randomized, double-blind, placebo-controlled trial on a mixed population of ataxia patients with different forms of hereditary cerebellar ataxia demonstrated that the exposure to 100 mg/day riluzole alleviated the SARA scores in patients and did not reveal any life-threatening toxicity (Romano et al., 2015). Thus, the research on the murine models and clinical trials on subjects with ataxia showed the potential therapeutic effect of riluzole and other SK and BK channels positive modulators.

In this study we performed long-term i.p. injections with CHZ to test if positive modulation of calcium-activated potassium channels can exert a beneficial effect in treatment of ataxic symptoms in HD. We firstly observed that CHZ treatment alleviated the age-dependent dysfunction in the firing pattern of 12-month-old YAC128 PCs (Figure 2). Thus, the recovery of the SS firing frequency was observed in YAC128 PCs after long-term CHZ injections (Figure 2B). The restoration of PC firing precision was also determined in YAC128 CHZ mice. The firing variability significantly decreased (Figure 2E) and the percentage of tonic cells significantly increased (Figure 2F) in YAC128 mice after treatment with CHZ. Injections with CHZ did not affect the CS firing frequency in any group (Figure 2C). However, they did recover the average mean of the post-CS pause in YAC128 mice (Figure 2D), thus indicating the improvements in the PF-PC synaptic transduction.

We further discovered that the CHZ treatment improved the CS duration in YAC128 mice (Figure 3B), but also induced changes in the spikelet frequency (Figure 3C). No effect on the spikelet number was observed in any group after injections with CHZ (Figure 3D). We assume that the improved CS duration may be observed due to the PC morphology improvement. Although the molecular layer thickness was similar in all groups tested (Figure 4B), we observed the recovery of the CF terminal localization in YAC128 mice after CHZ treatment (Figure 4C). We assume that the improved CS duration can be explained by the stronger spine formation in the YAC128 CHZ PCs, so the CF terminals discharge was processed better with those PCs. We suppose that the CHZ treatment improves the PC firing and morphology, but most likely does not affect the functioning of the inferior olive, the CF source, that may explain the observed changes in the spikelet frequency and spikelet number in YAC128 CHZ mice (Figures 3C,D).

Previously, CHZ treatment has demonstrated the improvement in motor activity in murine model systems of EA2 (Alvina and Khodakhah, 2010a) and SCA1 (Bushart et al., 2018). Being a FDA-approved drug, CHZ has very little amount of side effects in humans (Ernstgard et al., 2004). In our experiments, we observed a significant improvement in the motor activity of YAC128 mice after long-term CHZ injections, while treatment with this activator of calcium-activated potassium channels had no effect on the WT mice motor activity (Figure 6). Thus, we concluded that the use of CHZ may exert a potential therapeutic effect to treat the ataxic symptoms in HD.

The neurological phenotype of YAC128 HD mice includes the impairment of motor activity and striatal neuronal loss (Slow et al., 2003). Brain weight and striatal volume are significantly reduced in YAC128 HD mice compared to WT littermates since 9 months of age (Slow et al., 2003). Cortical volume is significantly less in 12-month-old YAC128 HD mice compared to WT mice of the same age, while no difference in cerebellum weight is observed at any age (Slow et al., 2003). YAC128 HD mice suffer from a motor deficiency detected by the rotarod (Slow et al., 2003) and beam-walk (Chen et al., 2011) tests starting at 6 months of age. Striatal neuronal loss and rotarod abnormalities are considered to be the primary neuropathological and behavioral endpoints for the YAC128 HD mice (Ehrnhoefer et al., 2009). The open-field analysis revealed that YAC128 HD mice develop hyperactivity at the age of 3 months, and display the hypokinetic behavior by the age of 12 months (Slow et al., 2003). YAC128 HD mice exerted very mild cognitive dysfunction in a simple swimming test, the time to reach the platform was significantly slower only in 8-month-old YAC128 mice compared to their WT littermates, while at the age of 10 and 12 months no significant difference between groups was detected (Van Raamsdonk et al., 2005). The pre-pulse inhibition was significantly decreased in 12-month-old YAC128 HD mice, while the habituation to acoustic startle was reduced in these mice only in the last block of pulse-alone blocks (Van Raamsdonk et al., 2005). Thus, the decline in cognitive functions observed in YAC128 HD mice is very mild.

Striatal neuronal loss is accompanied in YAC128 HD mice with age-dependent deficiency of dendritic spines in striatal medium spiny neurons (MSNs) (Wu et al., 2016). This spine loss may be explained by the calcium dysregulation in affected neurons (Miller and Bezprozvanny, 2010). In the previous studies with a model system of lipid bilayers it has been shown that the mutant huntingtin (mHtt), unlike WT Htt, binds to the C-region of the inositol 1,4,5-trisphosphate receptor (IP3R) on the ER membrane leading to its activation with a following enhancement of the calcium release from the intracellular calcium stores (Tang et al., 2003). The decrease of calcium level in the ER provokes the enhancement in the neuronal store-operated calcium (nSOC) entry in YAC128 MSNs (Wu et al., 2011). Knock-down of IP3R expression or the inhibition of nSOC normalized calcium status of YAC128 MSNs and recovered the spine density in these neurons (Wu et al., 2016). Thus, the disturbed calcium signaling in the affected neurons may be a cause for spine loss and the disease development.

The morphology of the cerebellar PCs has not been analyzed yet in the YAC128 HD mice, while the PC dysfunction can contribute into the motor deficiency of these mice. The loss of the PC dendritic spines is observed in many diseases associated with the cerebellar degeneration and motor decline. The reduced density of PC spines was reported for mice lacking βIII spectrin (Stankewich et al., 2010; Gao et al., 2011). This protein is highly expressed in PCs and stabilizes the glutamate transporter EAAT4. Mutations in βIII spectrin cause SCA5 disease. The mouse model of autosomal recessive spastic ataxia of Charlevoix-Saguenay also exhibited the reduced number of PC dendritic spines (Ding et al., 2017). The IP3R1 was reported to be crucial for the regulation of the PC spine density in mice (Sugawara et al., 2013). Meanwhile, the enhanced activity of IP3R was detected in different models of neurodegenerative disorders, including HD, SCAs, and Alzheimer’s disease (Egorova and Bezprozvanny, 2018; Hisatsune et al., 2018). Thus, in the previous studies with a model system of lipid bilayers it has been shown that the mutant ataxin-2 and ataxin-3, unlike WT ataxin-2 and 3, binds with IP3R on the ER membrane leading to its activation with a following enhancement of the calcium release from the intracellular calcium stores (Chen et al., 2008; Liu et al., 2009). Presumably, the constantly increased concentration of intracellular calcium activates SK channels and the resulting membrane hyperpolarization slows down the PC spikes generation (Meera et al., 2016, 2017). Meanwhile, the allosteric modulation of SK channels restores the precision of PCs pacemaking activity in different murine models of ataxia (Walter et al., 2006; Alvina and Khodakhah, 2010b; Shakkottai et al., 2011; Kasumu et al., 2012a; Egorova et al., 2016). We assume, that the pharmacological activation of SK channels enhances the hyperpolarization of the PC membrane and negatively modulates the activity of the voltage-dependent calcium channels thus causing the reduction of the calcium entry to the cytoplasm from the extracellular space (Egorova and Bezprozvanny, 2019).

In our experiments we observed less density of the dendritic CF-PC spines in YAC128 HD mice compared to their WT littermates, while the treatment with SK activator CHZ improved the morphology of CF-PC synapses in YAC128 HD mice (Figures 4A,C). The molecular layer was equal for the YAC128 and WT mice (Figure 4B) that is being in accordance with the similar cerebellar weight in mutant and WT mice (Slow et al., 2003). We suppose, that the possible molecular mechanism associated with the improvement of the CF-PC spines’ density after CHZ treatment may be the normalization of calcium level in the PCs bodies through the activation of the SK channels, similar to the improvements observed previously in the experiment on the striatal MSNs in YAC128 HD mice after the normalization of their calcium status (Wu et al., 2016).

In HD the striatal part of the basal ganglia is primarily affected (Reiner and Deng, 2018). The progressive loss of the striatal neurons and projection inputs mirrors the HD neuropathological symptoms (Reiner and Deng, 2018). Thus, slight bradykinesia during the pre-manifest stage evolves into the akinesia and rigidity during the last stage (Reiner and Deng, 2018). The YAC128 HD model reveals mild cognitive symptoms, while the significant decline in motor learning and motor coordination skills is much more prominent and starts much earlier than observed cognitive dysfunction (Van Raamsdonk et al., 2005). Although the open-field analysis showed that YAC128 HD mice develop hyperactivity at the age of 3 months (Slow et al., 2003) and that is earlier than observed motor decline, by 6 months of age no disturbances in open-field habituation are observed in these mice, and only by the age of 12 months more severe cognitive symptoms are developed such as hypokinetic behavior and pre-pulse inhibition (Van Raamsdonk et al., 2005). Meanwhile, the motor skills decline develops in these mice by 6 months of age and worsens progressively (Slow et al., 2003; Van Raamsdonk et al., 2005; Chen et al., 2011).

Basal ganglia are involved in the control of the higher order cognitive functions and their dysfunction is observed in different neurobehavioral disorders (Riva et al., 2018). Recent studies have shown that the cerebellum controls not only the coordination of movements and motor activity, but also takes part in the processes of higher nervous activity such as attention, thinking, planning, and decision-making (Marek et al., 2018). Indeed, the significant decline of cognitive functions is observed in SCA2 patients (Rodriguez-Labrada et al., 2019). It was common to believe that the dysfunction of the basal ganglia or cerebellar projections to the cortical motor areas provokes the decline in the coordination of movements, and the impairment of the basal ganglia or cerebellar projections to the prefrontal cortical areas causes the cognitive decline (Leisman and Melillo, 2013). Recently more evidences of the strong interconnection between the basal ganglia and cerebellar pathways were given (Bostan and Strick, 2018). The neurologic and psychiatric symptoms observed in the neurodegenerative disorders most likely are caused as a result of a complex action of the disturbances in the basal ganglia, cortex, and cerebellum interconnections (Bostan et al., 2018). Thus, it is becoming difficult to distinguish that motor and cognitive damages observed in YAC128 HD mice are caused by the cerebellum or basal ganglia dysfunction, while most likely this decline is caused by the disturbances of the integrated network. Here, the YAC128 HD mice exhibited the crawling behavior during the late stages of the disease together with a general motor decline. The injections with the SK channels activator CHZ improved this behavior. We assume that the difficulties in Beam walk performance in YAC128 HD mice may be caused not only by the cerebellar damages, but also by the basal ganglia dysfunction.

Studies on rats have shown that SK channels play an important role in the control of striatal neuronal firing and LTD, thus effecting the goal-directed actions and habits (Hopf et al., 2010). In this research we focused on the cerebellar physiology and morphology in YAC128 HD mice, and their affection by CHZ, the study of the SK channels modulation effects on the striatal pathology should be carried out.

## Conclusion

Activators of calcium-activated potassium channels can normalize the firing rate of cerebellar Purkinje cells. The alleviation of PC firing activity leads to the improvements in the cerebellar cells morphology and to the recovery of the motor symptoms in many mouse models of ataxia. Here, we have demonstrated that it is the case in HD mouse model too. The long-term i.p. injections of CHZ recovered the precision of PC pacemaking, improved the cerebellar Purkinje cells morphology, and also restored the normal motor performance in YAC128 HD transgenic mice. Thus, we propose the use of CHZ as a novel treatment of ataxic symptoms in HD. These results also suggest that similar approaches maybe used for symptomatic treatment in HD and ataxias.

## Data Availability Statement

All datasets generated for this study are included in the article/Supplementary Material.

## Ethics Statement

The animal study was reviewed and approved by the Bioethics Committee of the Peter the Great St. Petersburg Polytechnic University at St. Petersburg, Russia and followed the principles of European convention (Strasbourg, 1986; https://www.coe.int/en/web/conventions/full-list/-/conventions/treaty/123) and the Declaration of International medical association about humane treatment of animals (Helsinki, 1996; https://www.wma.net/what-we-do/medical-ethics/declaration-of-helsinki/doh-oct1996/).

## Author Contributions

PE and AG performed electrophysiological recordings and behavioral experiments and analyzed the data. PE wrote the manuscript. IB initiated and supervised the research, and prepared the final version of the manuscript for publication.

## Conflict of Interest

The authors declare that the research was conducted in the absence of any commercial or financial relationships that could be construed as a potential conflict of interest.

## Footnotes

1 https://www.coe.int/en/web/conventions/full-list/-/conventions/treaty/123

2 https://www.wma.net/what-we-do/medical-ethics/declaration-of-helsinki/doh-oct1996/
